# How COVID-19 Triggers Our Herding Behavior? Risk Perception, State Anxiety, and Trust

**DOI:** 10.3389/fpubh.2021.587439

**Published:** 2021-02-15

**Authors:** Yi-Chih Lee, Wei-Li Wu, Chia-Ko Lee

**Affiliations:** ^1^Department of International Business, Chien Hsin University of Science and Technology, Taoyuan, Taiwan; ^2^Department of Administration and Insurance Management, Cheng Ching General Hospital and Cheng Ching Medical Care System, Taichung, Taiwan; ^3^Feng Chia University Ph.D. Program in Business, Feng Chia University, Taichung, Taiwan

**Keywords:** COVID-19, risk perception, state anxiety, herding effect, trust in social media

## Abstract

People have felt afraid during the outbreak of coronavirus disease 2019 (COVID-19), because a virus is an invisible enemy. During the pandemic outbreak, society has become worried about the spread of infections and the shortage of protective equipment. This common fear among the public subsequently deepens each person's fear, increasing their belief in the content reported by the media and thus actively compelling these individuals to engage in the behavior of panic buying. In this study, we explored the effects of the public's risk perception, state anxiety, and trust in social media on the herding effect among individuals. The study was based on an online questionnaire survey and convenience sampling. The results showed that the public's risk perception increased their state anxiety and then deepened their willingness to wait in line for a purchase. In addition, the more people that trust the message delivered by the media, the more actively they will join the queue to buy goods. This study also found that anxiety had a greater impact on the public's willingness to wait for a purchase than trust in social media. Therefore, the top priority for the government should be to reduce the public's state anxiety and then reduce the herding effect.

## Introduction

In December 2019, the city of Wuhan detected cases of viral pneumonia with uncertain causes. On December 31, 2019, the World Health Organization (WHO) was informed that the Chinese government had discovered more than 40 cases of a new viral infection in Wuhan. The virus was a type of coronavirus that had not been previously identified. Trade between Taiwan and China was frequent. At the end of January 2020, Taiwan confirmed its first case of coronavirus disease 2019 (COVID-19) (1).

When a new disease appears, the public lacks adequate knowledge of and misunderstands the disease. They will perceive the new disease based on their previous experiences with the flu or other illnesses, which will influence their attitudes and preventative responses to the new disease (2, 3). COVID-19 is new virus, and the number of confirmed cases (and viral spread) are increasing rapidly. The coronavirus pandemic is filled with uncertainty (4). People become afraid during an outbreak of disease because a virus is an invisible enemy. No one can be sure if they are infected before the appearance of symptoms. However, once one has symptoms, it is considered “too late” (5).

Chaiken (6) proposed that as more people believe a message is correct then they agree that it is probably valid. This is a simple decision-making rule for individuals' cognitive processes and evaluations of information. According to the theory of the “bandwagon effect,” when individuals are guided by groups or encounter group pressure, they tend to question their own judgment and change their views or behaviors to share the same opinions as others (7). According to scholars, when people lack sufficient information and cannot make the appropriate choice, they tend to be influenced by others. For the public, although an individual's information may be insufficient, if the information between groups is gathered, the answer can be obtained. Therefore, in specific situations, solutions from the group can seem like the right choice. When individuals encounter a crisis and must make choices immediately, they will imitate others. With the spread of the media, this process results in a mass movement (8, 9).

Trust is the key factor to understanding these individual behaviors (10). Media trust includes three types: trust of news content, trust of news reporters, and trust of news corporations, which are trust in person-to-content, trust in person-to-person, and trust in person-to-system. Thus, media trust is a compound concept (11). People's judgements and evaluations of immediate or persistent threats to their health from the external environment are called “environmental risk perception” (12). Slovic (13) indicated that by following media reports, the public can recognize social risk issues and expand their social participation. Nevertheless, the public might overestimate the impact of risks (14). The media is significantly influential in disseminating risk information and influencing public perception.

The media's choice of risk issues and the degree of emphasis on their reports are highly associated with the audience's risk perception and risk acceptance (14). In a highly uncertain environment with complicated risk issues, the public acquires information based on media sources. Trust in media plays as a key factor in the judgment of risk (15). According to scholars, media reports can reinforce the public's risk perception of incidents. The media is the primary source for the public risk perception of specific events (13). In addition, emotion and human feelings are considered to be the important factors for understanding risk perception. Fear is an important emotional expression (16). The research revealed that respondents with low disease prevalence had higher environmental risk perception, and these individual's fear of the epidemic was also higher. These respondents also agreed that the severity of the disease was related to the risk of infection (12). Thus, risk perception is highly associated with emotion.

When people fight diseases, the public's information sources come from ongoing reports by the media. As the public is anxious about being infected, they will actively pay attention to the media messages related to themselves and value the accuracy of the messages (17). While some groups of people depend on governmental information to take precautions to prevent infection, others rely on related reports in social media to take precautions (17). Therefore, even though the government constantly assured a sufficient availability of necessary materials during the COVID-19 pandemic (18), many people still gave up their ideas in a group atmosphere and adopted a decision-making model consistent with those they observed to undertake panic buying (19). Thus, in China, Hong Kong, Singapore, and Taiwan, the public fought for and stocked up on masks, alcohol for disinfection, toilet paper, and even instant noodles, which significantly influenced their daily lives (20–23).

Previous studies on herding effects focused on investing in the stock market (24, 25), herding behavior in online peer-to-peer lending (26), and consumers' herd consumption behavior (27). Research on herding behavior in public health has been limited. Based on the above, this study aimed to understand whether risk perception, state anxiety, and trust in the media influenced public pandemic prevention behavior.

### Research Model

Le Bon (28) noted that when a certain number of groups gather for a certain action, they form new psychological characteristics. Under certain conditions (and only under those conditions), people who gather in groups present new characteristics. Unlike the original characteristics of individuals, like their occupation and gender, these new characteristics tend to make a person's concepts and ideas consistent. As a result, self-conscious personalities gradually disappear, while crowd psychology is formed, which is temporary but leads to real action. Under the influence of the COVID-19 pandemic (i.e., an occasional event), the public gathered together and rushed to purchase materials, thus producing these mass characteristics. The important characteristics of this psychological group, however, are temporary.

Regardless of whether the group members' individual lifestyles, characteristics, and intelligence levels are the same, being in a group results in the group's individuals forming a crowd psychology that produces widely different emotions, opinions, and behavior patterns among the public. In a group, all emotions and behaviors are contagious. As the group always pays attention to something with certain expectations, hints can easily affect change. In this situation, after the hint of a material shortage was transmitted to each member of the group (for example, through the media), this hint rapidly entered the individuals' brains and made the members' attitudes more consistent, thus producing artificially established facts and compelling the members to act.

During the outbreak of the pandemic, the public are, as a group, worried about infection and the shortage of protective equipment. This common fear among the public deepens each person's fear, increasing the public's belief in the content reported by the media and, thus, actively compelling these individuals to engage in the behavior of panic buying. Group emotions tend to function via extremes: extremely simplified or extremely exaggerated. If the group holds exaggerated and/or simplified attitudes about a matter, individuals in that group will rarely doubt or hesitate when acting on that matter. This factor is often related to negative emotions. Even though the public has managed to buy their necessary goods, they still feel inadequate and are thus compelled to continue purchasing related products under a negative feeling of fear.

According to the above factors affecting crowd psychology, this study uses trust in the media as the external factor to explore how public concern regarding the external environment changed via the media during the pandemic. The other two variables, risk perception and state anxiety, are manifestations of individuals' internal attitudes and emotions. In the end, people's irrational behaviors can result in a herding effect. For people under the condition of collective anxiety, risk perception might play an important role in affecting their behavior. Therefore, in this study, we argue that risk perception has a direct impact on herding behavior. A high level of risk perception is usually associated with people's inner states of anxiety. As a result, risk perception might have an indirect effect on herding behavior through increased state anxiety.

**Figure 1:**
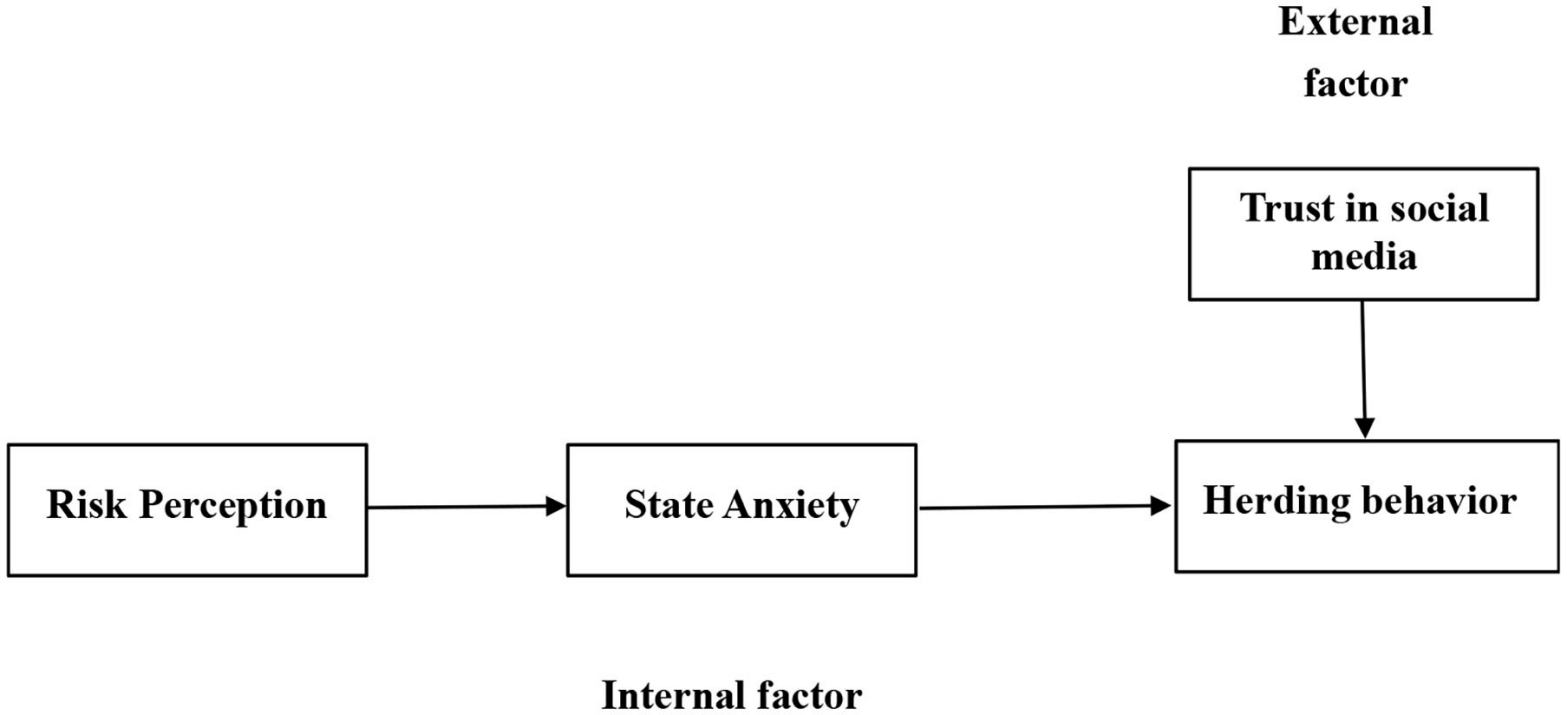
Research model.

Figure 1 shows the research model for the four constructs and their theoretical relationships, as discussed above.

### Hypotheses

Schüler and Nakamura (29) stated that perceived risk indicates an individual's emotional and cognitive responses to the possibility of a significant perceived loss. In addition, scholars observed that perceived risk directly influences consumer behavior (30). From a psychological perspective, when purchasing items, consumers are stimulated by the atmosphere and situation and evaluate the stimulation accordingly. When they perceive that they are threatened by this stimulation, they become anxious about the situation. This anxiety is called state anxiety (31).

Herding behavior is viewed as a behavior that irrationally and emotionally follows the behavior of a crowd (27). Lee and Wu (32) argued that different kinds of emotion influence consumer behavior. This study suggests that due to the global outbreak of COVID-19, individuals perceive their health as highly threatened. They attempt to lower their risk through appropriately responsive behavior. In addition, they see the panic buying of other people in stores, which triggers their own feelings of anxiety and stimulates them to act like others. Frewer (33) noted that information proven to be correct, professional, and unbiased has a positive impact on the public. The media and the public seek the opinions of experts through social media channels. The public does not know who to trust, but during an emergency, if the government intends to convey correct health advice, trust is the most important consideration (34). Therefore, in this study, trust in the media is defined as an individual's sense of dependence on the information transmitted by the media, willingness to take risks based on that information, and confidence in that information.

In this study, we deemed that when individuals show state anxiety in buying personal protective equipment and trust reports in the media, the result is that individuals will strengthen their queuing behaviors and produce the herding effect. An individual's own anxiety, thus, affects their behavioral judgments (35). Even if the public believes in the media's reports that there is no shortage of materials, when individual anxiety cannot be relieved, individuals still follow others and wait in line to purchase goods to reduce their anxiety. Therefore, individuals' real behaviors are influenced by their own internal emotions. Therefore, we propose the following hypotheses:

H1: An individuals' risk perception positively influences their state anxiety.
H2: An individuals' state anxiety positively influences the herding effect.
H3: An individuals' state anxiety is the mediator between risk perception and the herding effect.
H4: Trust in social media positively influences the herding effect.
H5: State anxiety has a higher impact on the herding effect than trust in social media.

## Materials and Methods

### Participants

This study was conducted during the period of the COVID-19 outbreak (February 20, 2020–March 20, 2020). We explored the public's psychological state and behavior in purchasing personal protective equipment during this period. The subjects included students from one university (including regular students and work-study students). The samples collected in this study were taken from those people who had the experience of lining up to buy personal protective equipment. Since the Ministry of Education of Taiwan made it mandatory for all students to wear masks in class, they used a very high number of masks every day. Therefore, it is appropriate to use students as the samples. This study was based on an online questionnaire survey and convenience sampling. We collected responses from 180 voluntary participants. Approval to participate in the online questionnaire denoted respondents' consent for involvement.

### Questionnaire

The questionnaire first asked about demographic information, including gender, age, family members at home, and recent frequency of purchasing personal protective equipment. In this study, personal protective equipment included surgical masks and alcohol or hypochlorous acid designed for public use. The questionnaire included four constructs, each measured using a 5-point rating scale from 1 (strongly disagree) to 5 (strongly agree). The herding behavior items were modified from Shantha (36) and were “I buy the masks by following others” and “I buy more expensive masks.” When the scores were higher, the respondents tended to follow others' behavior.

The risk perception items were modified from Lai et al. (37) and included “Do you think that you or your family members can be infected with COVID-19?” “What is the possibility that you or your family members are infected with COVID-19?” and “Can you or your family members be easily infected with COVID-19?” When the scores were higher, the perceived risk was higher. For anxiety, this study primarily measured state anxiety, including three items that were modified from Liao and Chen (31): “I worry that I cannot get the necessary products when lining up for personal protective equipment,” “I am not sure and I hope that I can get the materials,” and “I don't think that I can get the materials by lining up.” When the scores were higher, the subjects were more anxious about the situation.

The measured trust in social media indicates the public's trust in the information transmitted by online social media. For this, we used four items modified from Yang and Wang (38): “The information transmitted to the social media is objective,” “the information transmitted to the social media is not false,” “I think the information transmitted to the social media is correct,” and “in my opinion, my friends trust the information transmitted to the social media.” When the scores were higher, the public's trust was more substantial.

### Data Analysis

After the invalid measurement questionnaires were excluded, this study used the Statistical Package for Social Sciences (version 21.0 IBM SPSS Inc., Chicago, IL, USA) to perform statistical analyses. The average mean, standard deviation, and percentages were used in the descriptive statistics. The statistical methods included a Pearson correlation analysis and multiple linear regression to test the contribution and significance of the variables. This study also measured the Cronbach's alpha, composite reliability (CR), and average variance extracted (AVE) of each variable to understand the reliability and the validity of the scale. We used G-Power to calculate the sample size. The Kolmogorov-Smirnov test was used to test for normality of the distribution. We then used PROCESS Macro for SPSS, developed by Hayes (39), to verify the mediation effects. During the testing of the PROCESS Models, we used 10,000 bootstrap samples with 95 percent confidence intervals for the bootstrap analyses.

## Results

This study required at least 119 people for the main analysis, based on the power of 0.95 and alpha set to *p* < 0.05. We collected 180 valid samples. To test the reliability and validity of the questionnaire, this study first conducted an analysis using Cronbach's α. A value of 0.70 or higher indicated good reliability (40). The study conducted a validity analysis using composite reliability (CR) and average variance extracted (AVE). This study achieved reliability coefficients for most constructs higher than 0.7 (except herding bias) and AVEs higher than 0.5 (41). The Kolmogorov-Smirnov test showed that our sample match exhibits a normal distribution. In this study, the fitness of the measurement tool was generally positive (Table 1).

**Table 1:** The reliability and validity of this model.

**Variables**	**Cronbach's **α****	**Factor loadings**	**CR**	**AVE**
Risk perception	0.918	0.911–0.951	0.9481	0.8590
State anxiety	0.874	0.851–0.928	0.9228	0.7996
Herding bias	0.689	0.796–0.859	0.8134	0.6857
Trust in social media	0.827	0.757–0.864	0.8857	0.6599

*Composite reliability (CR) and average variance extracted (AVE)*.

This study explored the public's perception of waiting in line to purchase personal protective equipment and the recent increased demand of personal protective equipment due to COVID-19. We collected 180 valid samples from individuals waiting in line to purchase personal protective equipment during the pandemic, including 54 males (30%) and 126 females (70%); their average age was 23.4 (SD = 7.7, range = 18–55), and 59 subjects (32.8%) had at least one child at home below the senior year of high school. Fifty-one subjects (28.3%) had an elderly person above 65 years old living with them. In response to a question about “buying masks by waiting in line due to the pandemic situation in the past week,” 68 subjects (37.7%) said they had done so, including 49 females (72.1%); For “buying disinfection products, such as alcohol,” 65 subjects (36.1%), including 44 females (67.7%), reported that they had done so. Of those who purchased such materials, the majority were women. Less than half of the subjects reported buying the materials (Table 2). Table 3 lists the state anxiety scores, risk perception scores, trust in social media scores, and herding behavior scores according to gender and age groups.

**Table 2:** The sample characteristics.

**Variables**	**Values**
Gender	
Female	126 (70%)
Male	54 (30%)
Age (years)	23.4 (SD = 7.7/Range = 18–55)
Living with children	59 (32.8%)
Living with aging parents (≧65)	51 (28.3%)
I waited in line to buy face masks last week	68 (37.7%)
I waited in line to buy alcohol last week	6 5(36.1%)

*Number (Percentage)*.

**Table 3:** Constructs' scores according to gender and age.

	**Risk perception**	**Herd bias**	**State anxiety**	**Trust in** **social media**
Female	3.88 (0.68)	4.31 (0.73)	4.57 (0.60)	4.71 (0.56)
Male	3.81 (0.65)	4.26 (0.78)	4.41 (0.72)	4.69 (0.53)
Age ≧ 30	4.00 (0.51)	4.66 (0.71)	4.75 (0.33)	4.52 (0.46)
Age ≧ 20~<30	3.87 (0.68)	4.22 (0.72)	4.48 (0.68)	4.71 (0.50)
Age<20	3.74 (0.72)	4.30 (0.77)	4.50 (0.62)	4.80 (0.70)

*Mean (SD)*.

This study first validated the correlation between risk perception, state anxiety, and the herding effect. Using a regression analysis, we aimed to discover if state anxiety was a mediator. According to Table 4, there was a positive correlation between risk perception and state anxiety; this indicates that when the public's risk perception was higher, their state anxiety relating to the purchase of personal protective equipment was also higher and statistically significant (*p* < 0.05). Table 4 shows the correlation coefficients of the relationships between risk perception, state anxiety, and the herding effect. The coefficients all demonstrated a positive and significant correlation. Thus, when the public's perception of risk was higher, they became more anxious about waiting in line to buy personal protective equipment and tended to follow others to line up. The paired correlation coefficients between risk perception, state anxiety, and the herding effect were generally significant.

**Table 4:** The correlations between constructs.

**Variables**	**1**	**2**	**3**	**4**
Risk perception 1	1			
Herd bias 2	0.357^**^	1		
State anxiety 3	0.300^**^	0.570^**^	1	
Trust in social media 4	0.192^**^	0.253^**^	0.359^**^	1

** *p < 0.01*.

Next, we validated the hypotheses of the overall model. Based on the results, the standardized regression coefficient of risk perception on state anxiety was significant (β = 0.297, *p* < 0.001), and H1 was supported. The standardized regression coefficient of state anxiety on the herding effect was also significant (β = 0.572, *p* < 0.001), and H2 was supported. This study proceeded to test the mediator effect. We first assessed the risk perception and then state anxiety to examine their influence on the herding effect. The β of risk perception remained significant, albeit slightly lowered (β = 0.177, *p* = 0.011). The β of state anxiety was also significant (β = 0.519, *p* < 0.001). We also examined the predictive effects of the overall model. The F-tests of the two models were significant. In Model 1, the total variance of the herding effect explained by risk perception was 10.2, and in Model 2, this increased to 34.1% (increasing by 23.9%). This shows the mediating effects of state anxiety.

According to the statistical results, risk perception was able to positively predict the public's herding behavior. However, when state anxiety was included, the predictive capacity of risk perception was lowered but still significant. State anxiety was significant. Thus, risk perception and state anxiety were able to predict the public herding effect. State anxiety was the mediator, and H3 was supported. The results of the bootstrapping analyses conducted by PROCESS (Model 4) also supported this hypothesis. This study demonstrated that the indirect effect of risk perception on herd bias via state anxiety was 0.1709, with a 95% confidence interval that did not contain zero (CI = [0.0770, 0.2858]).

This study verified the relationship between trust and the herding effect. Table 4 shows that trust in social media and the herding effect were positively correlated, which indicates that the more people there are who trust in social media information, the more distinct the people's engagement in herding behavior will be. The standardized regression coefficient of trust to the herding effect was also significant (β = 0.252, *p* = 0.001), so H4 was supported. We then compared two variables, external trust and internal state anxiety, to determine which one had a greater impact on the public. The results showed that the public's internal anxiety had a greater impact on herding behavior than external trust in social media (β = 0.572 vs. β = 0.252); thus, H5 was supported. Please see Table 5.

**Table 5:** A summary of results.

**Hypotheses**	**β value**	***p*-value**	**Verification**
Risk perception → State anxiety	0.297	*p* < 0.001	H1 was supported
State anxiety → Herding behavior	0.572	*p* < 0.001	H2 was supported
Risk perception → State anxiety → Herding behavior	0.1709	*p* < 0.05	H3 was supported
Trust in social media → Herding behavior	0.252	*p* = 0.001	H4 was supported
State anxiety → Herding behavior > Trust in social media → Herding behavior	0.572 > 0.252	*p* < 0.05	H5 was supported

## Discussion

This study mainly explored the Taiwanese people's attitudes toward the purchase of personal protective equipment related to COVID-19. First, we attempted to determine if risk perception and state anxiety caused the public to line up to purchase these products. According to the results, public perception of a high risk of COVID-19 strengthened their desire to buy personal protective equipment by waiting in line. In addition, the public's temporary anxiety in the decision-making situation reinforced their intention to line up. Using a regression analysis, this study sought to discover if state anxiety was a mediator between the public's risk perception and their lining-up behavior. Based on the findings, risk perception was critical. When people were more anxious, they were more likely to wait in line to purchase personal protective equipment.

COVID-19 is a new contagious disease, and, at present, effective medicine to fight it is lacking. In addition, the numbers of confirmed cases and deaths in different countries are increasing. Thus, the public's perceived risk of COVID-19 remains high. Although epidemic prevention materials continued to be produced, the public still worry that they will not be able to obtain such products. Therefore, regardless of the results of success in purchasing such equipment, these individuals continue lining up due to anxiety. This result presents the herding effect of the personal protective equipment.

This study also compared the impact of state anxiety and trust in social media on people's lining-up behavior. The results showed that when people line up to buy personal protective equipment, their fear of being unable to buy the necessary materials and their mentality that they will be unable to buy enough materials together strengthened their behavior of lining up with the intent to purchase, even suggesting to their relatives and friends that they also buy such materials. This is also the main reason why, in many countries, there were often long queues in drug stores or supermarkets. At the same time, individual state anxiety had a much higher impact on queuing behavior than trust in media reports. In other words, an individual's internal anxiety exerted a greater influence than trust in media reports and was also a key factor affecting people's queuing behavior. Therefore, reducing the public's state anxiety should be a priority for the government.

As the pandemic situation escalated in other parts of the world, the shortage of personal protective equipment turned more severe. According to this study, risk perception, state anxiety, and trust in the information on social media influenced the public's willingness to line up and buy said equipment. State anxiety, however, is the primary factor. One possible solution is to use information technology to help effectively manage public state anxiety. Such technology can be used, for example, to construct purchasing maps of personal protective equipment. In addition, technology can be used to schedule times for sales, determine stock availability, and even for reservation systems. At present, almost everyone has a phone, and by using online maps, the public can acquire the necessary information to successfully purchase personal protective equipment. The government or shops can collect the sales records of personal protective equipment and use that information to plan subsequent delivery. The public can identify the available stock of masks or alcohol in different shops online. In this way, the public can be prepared and avoid state anxiety. Thus, technology can reduce disputes between the public and the shops, and people can avoid wasting their time waiting in line.

Human behavior has a tendency toward “loss aversion.” When losses are equal to gains, people are more sensitive to the losses than they are to the gains. Therefore, effectively reducing the public's panic regarding the disease, the Taiwanese centers for disease control live-broadcast the latest pandemic information both at home and abroad during a fixed period of time every day. The purpose of these broadcasts is to enhance the public's trust in the public health policies issued by the government by reporting the results of actual pandemic prevention measures in an open and transparent manner. The media also have a responsibility to spread the truth and should avoid making inflammatory reports that might trigger mass hysteria. In addition, the government also requires that people maintain compulsory social distancing or wear a mask if proper social distancing cannot be maintained to protect the safety of the public (42). Due to the high degree of public cooperation, the number of cases has been reduced, as has the public's risk perception of the disease, sense of anxiety, and the number of people lining up for panic-induced purchases at drug stores and supermarkets. This research framework demonstrates that the government can take appropriate measures to reduce the herding behavior of the public.

### Limitations

There are many factors that affect the fight against the epidemic. This study explored the public's situation-based issues and behavior when purchasing personal protective equipment (masks and alcoholic hand sanitizer). In addition to personal protective equipment, the public may also need to fight for the necessities of life. We suggest that future researchers include such daily necessities in their research. In addition, this study did not explore if the public waiting in line used information technology to inform their purchasing behaviors. The effect of new technology on such behaviors can also be studied in the future. For the herding effect, the Cronbach's alpha was 0.689, and although it was reasonable (43), we suggest that researchers use these items more carefully. As Taiwanese people tend to feel awkward in admitting that they have lined up with others to buy personal protective equipment, it was somewhat difficult to collect relevant samples of such actions. Moreover, the disadvantages of online surveys are non-response error and limited sampling. Thus, this study's sample size is small. As most samples were from young age groups, this study also suffers from inferential limitation. We suggest that researchers further investigate and compare different age groups in regards to this research topic.

## Conclusion

The results of this research revealed that risk perception, state anxiety, and trust in social media trigger herding behavior, with state anxiety being the most important factor to induce such behavior. Taiwan is one of few countries where people can still go to work and school normally during the COVID-19 pandemic. It is already considered polite to wear a mask when sick in Taiwan. The government has intervened in the distribution and production of personal protective equipment since the beginning of the outbreak. However, aspects of the pandemic situation remain highly uncertain. Confirmed cases may or may not show symptoms. For self-protection, the public may fight over personal protective equipment for their safety. With limited resources, the government should take effective actions to actively protect the safety of frontline medical personnel and the public. This will instill confidence in the ability of the government's policies to successfully resist COVID-19.

## Data Availability Statement

The raw data supporting the conclusions of this article will be made available by the authors, without undue reservation.

## Ethics Statement

Approval to participate for an online questionnaire is consent for participation. Written informed consent for participation was not required for this study in accordance with the national legislation and the institutional requirements.

## Author Contributions

Y-CL and C-KL: conceptualization. W-LW: methodology. Y-CL and W-LW: writing—original draft. All authors contributed to the article and approved the submitted version.

## Conflict of Interest

The authors declare that the research was conducted in the absence of any commercial or financial relationships that could be construed as a potential conflict of interest.
